# Functional Benefits of Brewer’s Spent Grain and the Challenge of Developing Food Ingredients for Human Health

**DOI:** 10.3390/antiox15020247

**Published:** 2026-02-13

**Authors:** Carmen Soto-Maldonado, Alejandra Espinosa, José Luis Bucarey, Lida Fuentes

**Affiliations:** 1 Centro Regional de Estudios en Alimentos Saludables (CREAS), Avenida Universidad 330, Placilla, Curauma, Valparaiso 2362696, Chile; carmensoto@creas.cl; 2 Pontificia Universidad Catolica de Valparaiso, Avenida Brasil 2950, Valparaiso 2340025, Chile; 3 School of Medicine, Faculty of Medicine, Universidad de Valparaiso, San Felipe 2172972, Chile; 4 Center of Interdisciplinary Biomedical and Engineering Research for Health, Universidad de Valparaiso, San Felipe 2172972, Chile

**Keywords:** ferulic acid, p-coumaric acid, arabinoxylans, diet fiber, bioprocessing, brewer’s bagasse, beer by-product, antioxidant action, anti-inflammatory action

## Abstract

Recent developments have highlighted the importance of using by-products such as brewer’s spent grain (BSG) as functional food ingredients. BSG is a source of polyphenols, antioxidants, dietary fiber, and protein, all of which are crucial for preventing metabolic syndrome and cardiovascular disease. This review focuses on bioactive compounds in BSG, such as ferulic and p-coumaric acids, which are distinguished for their antioxidant and anti-inflammatory properties. These compounds play a vital role in liver protection by boosting antioxidant enzyme activity and reducing inflammation and oxidative stress. Additionally, arabinoxylans (AX) in BSG enhance gut health by promoting the growth of beneficial microbiota. The review also identifies gaps in understanding how processing affects bioactive compound bioavailability in foods, emphasizing the need for innovative food technologies to enhance nutrient release and absorption, including the recent studies that show the role of gut microbiota in fermenting BSG components, leading to the release of beneficial compounds, such as ferulic and butyric, which positively influence health and metabolism. Despite these advancements, further research is essential to explore the interactions within the gut, liver effects, and the overall health benefits of BSG, aiming to optimize processing techniques to preserve its nutritional value and promote sustainable food innovation.

## 1. Introduction

Beer is a globally popular alcoholic beverage with China, the United States, and Brazil leading the market in production quantity [1]. While beer can be brewed from various cereals, barley is the primary malting grain used in most countries to produce brewer’s wort. This wort is fermented and matured to create beer. The brewing process, besides using water, produces several by-products, including spent grain, spent brewer’s yeast, and spent hops/hot trub [2]. The most significant by-product of this process is bagasse or brewer’s spent grain (BSG), which constitutes 85% of the residues and about 31% of the malt’s original weight [3]. The increase in beer production due to its widespread consumption has led to a rise in by-products, particularly bagasse [4]. In 2021, global beer production reached approximately 1.86 billion hectoliters, resulting in about 37.2 billion tons of bagasse [4]. It is estimated that for every hectoliter of beer produced, around 20 kg of bagasse is generated, making it a readily available resource [5,6].

Traditionally, BSG has been used as animal feed (cattle, cows, sheep, and fishes) due to its protein, fiber, and amino acid content, and its low cost [4,7,8,9,10]. Additionally, BSG can be used in the generation of biodegradable films and coatings [11]. On the other hand, BSG has been used in more advanced industrial processes such as direct combustion, biogas production, bioethanol production and composting [12,13,14,15]. However, biofuel production is an expensive process, requiring significant amounts of water and high energy costs [14]. Additionally, physicochemical characteristics such as humidity, C/N ratio, and pH range (optimum 5.5 to 7.5) may present disadvantages for composting process [15].

Several agricultural and industrial by-products such as rice, buckwheat, almond, pistachio hulls, banana, orange peel, and wine lees, as well as beer by-products, are rich in phenolic compounds, antioxidants, fiber, and other nutrients [16,17,18,19,20]. Citrus industry by-products, including peels and seed residues, also contain significant phenolic content [21]. By-products from the apple juice process [22] and pomegranate peel [23] have more phenolics than their edible parts. Olive mill waste and leaves are notable for their oleuropein content [24]. Grape and tomato seeds and peels have more phenolic compounds than their pulp [25,26,27]. Brewer’s grain has great potential as a rich source of polyphenols and soluble fiber [16,17]. Therefore, the valorization of by-products has the potential to address waste disposal issues and promote the development of innovative products that leverage the health benefits of nutrients and bioactive compounds.

Brewer’s bagasse is rich in important antioxidant compounds, including ferulic and p-coumaric acids, as well as other polyphenols [16]. However, despite its potential benefits, there are still several challenges to using Brewer’s bagasse as a food ingredient. Various technological processes such as supercritical extraction, solid liquid extraction with different pretreatments, fermentation processes, enzymatic or alkali and acid treatment prior to fermentation process and hydrothermal process, among others, can be employed to obtain valuable compounds such as arabinoxylans, proteins, ferulic acid, xylitol, xylose, and lactic acid, positioning brewer’s bagasse as a cost-effective and viable raw material for the production of sustainable bio-based products as a biorefinery strategy [28].

The knowledge gaps encompass not only the need to standardize the processes but also the necessity to understand how these techniques could affect the bioactivity of polyphenols and arabinoxylans (AX). Furthermore, in different foods, the assimilation of these polyphenolic compounds depends on interactions with fibers, principally AX [27,29,30]. Therefore, it is essential to develop methods to enhance the bioavailability of polyphenols, considering their association with fiber.

This review explores the chemistry, occurrence, bioavailability, potential uses of bioactive compounds, how processing can affect the bioavailability of bioactive molecules, and the knowledge gaps necessary to develop ingredients with higher antioxidant and fiber content from beer bagasse. With this aim, a comprehensive literature search was performed in PubMed, ScienceDirect, Scopus, Web of Science, and Google Scholar. The information has been collected since 2022. The study utilized combinations of the following stated keywords: “Ferulic acid,” “p-Coumaric acid,” “Arabinoxylans,” “Dietary fiber,” “Bioprocessing,” “Bioactive compounds”, “Biotechnology”, “Brewer’s bagasse,” “Beer by-product,” “High-fat diet,” “Antioxidant action,” and “Anti-inflammatory action.” The bibliographic corpus comprises significant references from 2002 to forthcoming studies, as well as recent articles up to 2025. The studies included looked at (a) the nutrition and chemical composition of beer bagasse, especially focusing on phenolic compounds (ferulic and p-coumaric acids) and dietary fiber; (b) research on processing and bioprocessing technologies (alkaline extraction, enzymatic extraction, ohmic heating, and microwave heating) intended to enhance bioavailability; (c) research assessing biological impacts on rodent models and the impact on human health. Exclusion criteria included (a) studies that only looked at non-food industrial uses (like biofuels) or studies without information about how to obtain bioactive compounds or how stable they are, and (b) articles that used a different biological model (like bacteria, fungi, etc.). The information was discussed based on an analysis of knowledge gaps and the potential for scientific and technological innovation in the use of BSG as a food ingredient.

## 2. Nutritional Composition and Physicochemical Characterization of BSG

Brewer’s bagasse is a raw material containing bioactive compounds such as phenolics and resistant starches [31,32,33,34,35,36,37,38,39]. However, the assimilation of an antioxidant in a complex food matrix depends not only on its content in food but also on the presence and interaction with other nutrients, the relationship between antioxidants and proteins and fiber, among other molecules, and the rheological characteristics of the food matrix [40,41,42,43]. Based on this, it is essential to know the nutritional composition to develop food from brewers’ waste (Table 1).

The moisture of the BSG after its industrial generation is very high (75–82%) [4,33] being necessary to remove this water for the stabilization of the sample. After drying in the oven, the malt bagasse bran had a moisture content of 4.4 to 5.6% [32]. Flour in general—wheat and wholegrain flour—have maximum moisture limits of 15% [34]. The low humidity of BSG after drying processing and the fact that its production in many parts of the world is associated with small breweries make logistics and the storage of raw materials more feasible. This reduces the risk of spoilage microorganisms growing, i.e., mycotoxigenic *Fusarium* spp., *Aspergillus* spp., and *Penicillium* spp., which is a requirement for developing safe ingredients from agro-industrial wastes [44].

The energy value of dried malt bagasse has been determined at 248 Kcal per 100 g, which falls into the high energy value range according to RDC Nº 54-Brasil [32]. The fat range reported is 5.9% to 7.69% [32,35,36], being about 50% essential fatty acids [43]. The ash corresponding to the mineral salts observed in dried malt bagasse bran ranges from 2.7 to 4.6% [32], showing zinc, sodium, iron, phosphorus, manganese, calcium, and potassium, highlighting that phosphorus and calcium are in more significant quantities [14,32].

Brewer’s bagasse is a by-product with high protein and fiber content, commonly used as animal feed and compost for soil nutrition [37]. The range of protein content for the dried malt bagasse bran has been reported from 12.5 to 17.9% [32,35,36], while the carbohydrate content is between 10% and 46.9% on a dry basis [32,35,38]. An advantage of beer bagasse as a food ingredient is that the fiber range for malt bagasse bran would be considered high, showing a range of 40–48% [32].

It should be noted that the interest in developing ingredients from BSG is also associated with the presence of bioactive compounds that can contribute to human health. The following section describes the antioxidant capacity, main antioxidant molecules, and other bioactive molecules whose interaction determines the release of antioxidant compounds; therefore, their assimilation and its effect on health are associated with its impact on liver protection and intestinal health.

## 3. Antioxidant Capacity and Phenolic Compounds Content in BSG

Brewer’s bagasse is a raw material rich in compounds with significant biological activity, making it highly valuable for food and pharmaceutical industries. It contains bioactive compounds such as phenolics, particularly ferulic acid, as well as resistant starches and arabinoxylans [31,32]. The presence of vitamins (thiamine, pyridoxine, niacin, and cobalamin) [45] and polyphenols in foods can significantly enhance their antioxidant capacity.

### 3.1. Phenolic Compound Content in BSG

Phenolic compounds are important secondary metabolites in plants and play a significant role in growth, reproduction, and protection against pathogens and predators [46]. They have various physiological properties, including antioxidant activity, and have been linked to health benefits from consuming fruits and vegetables [47]. Antioxidant molecules, such as vitamins (ß-carotene, tocopherol, ascorbic acid) and polyphenol compounds (curcumin, tannins, gallic acid, caffeic acid, ferulic acid, carnosic acid, p-Coumaric acid, quercetin, catechin), protect against cellular damage induced by free radicals, which have been studied by their potential benefits on non-communicable chronic diseases. These antioxidant molecules neutralize free radicals outside and inside cells, depending on their chemical structure and capacity to enter membranes [48,49,50]. These antioxidants can not only be consumed from foods such as fruits and vegetables [51], but it also presents in agro-industrial waste [52,53,54].

In barley, phenolic compounds are typically found to be associated with dietary fiber, occurring at total polyphenol content of 0.2 to 3.1 mg GAE g^−1^ (gallic acid equivalents on fresh weight) and in the specific case of total flavonoid content of 0.4 to 0.7 mg g^−1^ (quercetin equivalents on fresh weight), depending on the origin of the grains [55,56]. Specifically, it is recognized that barley grains contain anthocyanins, flavonoids (catechins), proanthocyanidin and phenolic acids. In this last group of compounds, protocatechuic, vanillic, gallic, syringic, p-coumaric, caffeic, ferulic, and sinapic acids have been observed, with ferulic and coumaric acids with the highest content (over 500 µg g^−1^) [57]. The above allows us to consider the phenolic compounds that can potentially be found in brewer’s grain. Studies have documented the presence of these compounds in BSG, revealing that Imperial Red Ale BSG has a total polyphenol content of 7.30 mg GAE g^−1^ (gallic acid equivalents on dry material) and Belgian Strong Ale BSG has 9.55 mg GAE g^−1^, with approximately 70% of these being bound phenolic compounds [16]. Another group showed lower polyphenol content: 1.70 mg of (GAE g^−1^) for unfermented BSG, 1.26 mg GAE g^−1^ for milled BSG, and 2.28 mg GAE g^−1^ for extruded BSG [58]. This research indicates that extrusion methods markedly increased the total phenolic compounds relative to untreated and milled samples [58]. On the other hand, it has been described that the total content of soluble polyphenols and flavonoids varies according to the time of treatment with ultrasound or fermentation with *Lactococcus lactis*, observing significantly similar values for an ultrasound of 30 min (polyphenols 0.86 ± 0.42 mg GAE g^−1^, flavonoids 0.14 ± 0.0 mg QE g^−1^) and fermentation of 48 min (polyphenols 1.30 ± 0.22 mg GAE g^−1^, flavonoids 0.14 ± 0.01 mg QE g^−1^), with respect to BGS without treatment (polyphenols 1.42 ± 2.2 mg GAE g^−1^, flavonoids 0.13 ± 0.0 mg QE g^−1^) [59]. However, the total polyphenol and flavonoid content decreases significantly under other treatment conditions [59].

A unique one-step hydrothermal extraction method utilizing acidic natural deep eutectic solvents at 120 °C has recently been devised for the extraction of polyphenolic chemicals from BSG and malt dust (MD), enhancing the extraction yields by as much as 381% for BSG and 251% for MD, relative to conventional procedures [60]. Different types of malts showed values ranging from 16.2 to 20 mg GAE g^−1^ BSG [61]; these differences observed can be due to the different types of malts used in brewing and the processing conditions. On the other hand, the concentration of polyphenols in residual brewing yeast averaged between 1.13 and 14.26 mg GAE g^−1^; however, the interaction between temperature and prior treatment significantly influences total polyphenol content, with air-dried samples extracted at 95 °C yielding optimal results [62].

Based on the above, phenolic compounds derived from brewer’s bagasse show significant variability in total polyphenol content, influenced by factors such as beer type, processing methods, and extraction techniques [16,46,47,58,60,61,62,63]. This underscores the importance of optimizing extraction techniques to maximize the recovery of valuable antioxidants from biomass waste.

### 3.2. Antioxidant Capacity of BSG

Over the last 10 years, the number of studies evaluating BSG antioxidant activity has increased. In the case of Imperial Red Ale BSG, the antioxidant capacity of dried samples, measured in Trolox equivalents (µM TE per g of dry matter), for BSG using different methods has yielded values of 31.20 µM TE g^−1^ (determined by ferric-reducing antioxidant power, FRAP), 15.31 µM TE g^−1^ (determined by 2,2-diphenyl-1-picrylhydrazyl, DPPH), and 40.10 µM TE g^−1^ (determined by 3-ethylbenzothiazoline-6-sulfonic acid, ABTS). In contrast, the Belgian Strong Ale BSG samples exhibited higher values of 42.13 µM TE g^−1^ (FRAP), 30.43 µM TE g^−1^ (DPPH), and 73.17 µM TE g^−1^ (ABTS) [16]. Likewise, Belgian Strong Ale BSG samples exhibited elevated antioxidant activity within the setting [16]. Similarly, freeze-dried BSG from a mixture of malts (pilsen, munich, and carahell dark) showed an antioxidant activity of 5.5 and 40.05 mg TE g^−1^ BSG for the DPPH and ABTS methods, respectively; these values decrease slightly after the supercritical fluid extraction process to levels of 4.21 and 37.60 mg TE g^−1^ BSG [63]. It is worth mentioning that BSG has a higher antioxidant activity than that observed in malt. The antioxidant capacity of bagasse obtained from different types of malts shows that the values range from 16.2 to 20 mg GAE g^−1^, with a radical scavenging capacity of 12 to 15% for the DPPH method and 31.7 to 35 mg TE g^−1^ DW for the ABTS method. Interestingly, the BSG obtained from light malts presents better results than those presented by dark malts, potentially due to the process temperatures (over 200 °C for dark malts) [61]. These findings highlight the potential of BSG as a valuable source of antioxidants, particularly for light malt varieties.

### 3.3. Content and Extraction of Bioactive Molecules from BSG

Among the antioxidant compounds described in brewer’s bagasse, ferulic and p-coumaric acids stand out; sinapic, caffeic, and syringic acids are also present [64]. Previous review describes in detail the average quantity of each phenolic compound extracted from BSG by different techniques [64]. In this sense, ferulic and p-coumaric acids can be extracted in higher concentrations, ranging from 73 to 477 mg per 100 g of bagasse, using methods such as ultrasound-assisted extraction [65], solid–liquid extraction [31,66], and alkaline hydrolysis [67]. In contrast, caffeic and syringic acids can be extracted at concentrations near to 12–14.2 mg per 100 g but these require microwave-assisted extraction or solid–liquid extraction methods [64,68]. Catechin and its derivatives, such as (+)-catechin and procyanidin B, have been detected by HPLC-DAD after acetone-based extraction, depending on the type of bagasse [69]. In addition, it has been described that the total content of proanthocyanidins varied from 540.04 µg g^−1^ to 1002.31 µg g^−1^ D.W of GSG, observing the detection of catechin/epicatechin, dimers, trimers, and tetramers by HPLC-FLD-MS analysis using an extraction of 75% acetone and Sonotrode Ultrasonic Extraction (400 W) [70]. Given the high yield of ferulic and p-coumaric acids from BSG, efforts to obtain ingredients from this by-product should focus on processes that preserve the stability and activity of these compounds.

In addition to molecules with antioxidant capacity, there are bioactive molecules derived from fiber that are important for health and are highly present in BSG [17]. Among them, AXs are important for health due to their role as dietary fibers that cannot be digested by human enzymes, offering various physiological benefits, enhancing gut health by promoting beneficial bacteria, such as bifidobacteria and lactobacilli, and having prebiotic properties [71,72].

#### 3.3.1. Ferulic Acid (FA) in BSG

Ferulic acid is found in a wide array of plant matrices, existing in free form, conjugated form—bound to sugars and low molecular weight molecules—and insoluble form as part of AX and lignocellulosic complexes [73]. Extraction of ferulic acid has been developed from various sources, including corn bran, rice bran oil, wheat bran, brewer’s spent grain, and tomatoes, among others [73,74,75], using different methods. These methods are primarily laboratory-based techniques, such as Soxhlet extraction, accelerated solvent extraction (ASE), microwave-assisted extraction (MAE), ultrasound-assisted extraction (USE), subcritical water extraction (SWE), and pressurized water and hydroalcoholic mixtures. Additionally, techniques used at an industrial level include alkaline hydrolysis [73]. The efficiency of these extraction techniques largely depends on the raw material and the form in which ferulic acid is present.

Solvent extraction techniques are effective for recovering free ferulic acid, while conjugated or insoluble ferulic acid requires processes with more extreme operating conditions, such as high temperature, chemical compounds (alkali or acids), and pressure [73,75]. Industrial recovery of ferulic acid is typically achieved through alkaline hydrolysis of oryzanol from rice bran oil soapstock, followed by solvent extraction, especially in rice-producing countries like India, China, and Japan [74]. For instance, applying an alkaline process to sugar beet pulp under conditions of 12 h at 41 °C with 2 M NaOH allows for the recovery of 1.29 g of ferulic acid per 100 g [76].

In the case of BSG, it has been reported that ferulic acid is mainly bound to cell wall structures [16], so a hydrolytic process is necessary for its recovery. Then, various laboratory-scale extraction methods have been reported, including solvent extraction, chemical extraction, supercritical extraction, and microwave-assisted processes, among others [77]. The alkaline hydrolysis (120 °C, 1.5 h, 2% NaOH—20 mL per 1 g of bagasse), followed by ethanolic and acidic processes for neutralization, demonstrated a maximum recovery of 476 mg of ferulic acid per 100 g of BSG [67]. However, despite the high release of ferulic acid, the use of alkaline and acid hydrolysis and harsh operating conditions limit the product’s application in food and pharmaceutical industries [78]. In addition, chemical hydrolysis results in a complex liquor, which can contain sugars, proteins, and other phenolic compounds, as well as unwanted compounds produced by the degradation of the previous molecules, and therefore several purification steps are required for its recovery [73,78]. An alternative that has emerged in recent years is enzymatic processes, where catalysts with carbohydrase activity hydrolyze the plant cell wall, facilitating the release of the compound of interest [73]. In this context, using an enzymatic process with pure feruloyl esterase on brewer’s bagasse allows for the recovery of up to 24% of the ferulic acid from a total of 245 mg per 100 g when a pretreatment in an autoclave is performed. When the enzymatic catalyst is performed using a commercial pool (i.e., Depol 740L, Biocatalysts Inc., Tampa, FL, USA) containing at least cellulase, xylanase, and feruloyl esterase, the recovery increases to about 45% [79], indicating the improvement in ferulic acid extraction.

#### 3.3.2. p-Coumaric Acid (pCA) in BSG

p-coumaric acid, or 4-hydroxycinnamic acid, is a phenolic acid found in various fruits, vegetables, and cereals. It is also a precursor to other phenolic compounds. p-coumaric acid is known for its antioxidant, anti-inflammatory, and antimicrobial properties.

Specifically, in the case of brewer’s grain, p-coumaric acid is present, like ferulic acid, in conjunction with the fiber structure. Given the above, its quantification requires its release from the plant structure. In addition, its content depends significantly on the type of raw material (barley origin and/or other ingredients incorporated into the malting process) and the processes used. Some authors report that the p-coumaric acid content in brewer’s grains ranges from 565 to 794 μg g^−1^ [77,80,81], while others report lower values, such as 488.51 μg g^−1^ [82]. Likewise, it is reported that this compound is present in a 1:4 molar ratio with respect to ferulic acid (p-CA:FA) [67].

Regarding p-coumaric acid recovery processes, although solvent extraction is the most traditional method for recovering phenolic compounds, recovery levels are low and highly dependent on operating conditions, with maximum recovery levels of 4.1 mg g^−1^ [83]. This can be improved by applying processes such as ultrasound and microwaves, or thermal, chemical or enzymatic treatments, which allow the breaking of the fiber structure that contains them. In this regard, it has been reported that it is possible to obtain a liquor with up to 97.2 mg L^−1^ of coumaric acid when using an alkaline process [84]. On the other hand, ultrasound and ethanol or methanol water mixtures for extraction show a recovery at levels of 0.12 mg L^−1^ [85]. For example, applying heat treatment prior to extraction with solvent and ultrasound allows the recovery of coumaric acid from 3.71 to 11.42 ug/g [86]. These values can also be increased to levels of 3371.9 ug/g when applying an alkaline solution as extraction mixture [66]. In cases such as the use of microwaves in conjunction with alkaline solutions (0.75% NAOH), recoveries of p-coumaric acid of up to 47 mg per 100 g of bagasse, and 149 mg per 100 g of ferulic acid, are observed [61]; a similar value with a recovery of 60 mg per 100 g with a 5 h alkaline treatment was also observed [87], while enzymatic processes did not allow the recovery of the compound of interest. On the contrary, applying a hydrothermal process increased recovery to 1400 mg/100 g values. This demonstrates the varied effects that extraction processes and their operating conditions can have on the recovery of these types of compounds.

#### 3.3.3. Arabinoxylans in BSG

Arabinoxylan is a non-starch polysaccharide present in the cell walls of cereals such as wheat, corn, rye, and oats, and by-products such as BSG [17,42]. It comprises a linear xylose backbone with arabinose substituted at various positions. AX serves as soluble dietary fibers that promote gut health by acting as prebiotics. These fibers reach the colon intact, where they are fermented by *Bifidobacterium* species, generating short-chain fatty acids (SCFAs) that support gut homeostasis, reduce inflammation, and enhance intestinal barrier integrity [88,89,90,91,92,93,94].

Its content in brewer’s bagasse is primarily related to the characteristics of the raw material. Various authors have quantified and reported the presence of arabinoxylans in brewer’s pomace, as such either the sum of arabinoxylans and xylose, or hemicellulose, considering that the latter is mainly composed of arabinoxylans. In this regard, it is possible to observe levels ranging from 165 to 309.5 g/kg [80,81,95,96,97,98]. In this case, it has been shown that the recovery of these compounds from brewers’ grains depends primarily on raw material–particle size and the recovery processes. These include enzymatic processes using commercial products with xylanase activity, which allow for the recovery of up to 33% of soluble arabinoxylans [95]. The combination of enzymatic processes, but in this case for starch removal, heat treatment, and chemicals, has also been evaluated to recover arabinoxylans, achieving yields close to 40% of the raw material content [82]. The use of techniques such as solid substrate fermentation, ionic liquids, and enzymatic processes, in a refinery strategy, have made it possible to obtain products with 26.59% xylan and 7.04% arabinan, from an original matrix with 19.14% xylan, and 9.82% arabinan [99]. Regarding particle size, its variation primarily influences the determination of xylan content. Generally, it is observed that larger particle sizes correspond to higher xylan content [100].

As previously mentioned, ferulic acid is closely linked to arabinoxylan structures, which leads to its low bioavailability. Consequently, it is essential to recover both ferulic acid and arabinoxylans. Additionally, certain sources of arabinoxylans, such as wheat bran, can increase the nutritional value of products made from these raw materials, but can negatively impact the organoleptic qualities, such as the texture [101]. An *in vitro* digestibility assay revealed that wheat fractions and breads have less than 1% bioaccessibility of ferulic acid (FA) but adding free FA to flour boosts bioaccessibility to about 60% [102]. This suggests that bioaccessibility is significantly affected by the proportion of free FA, as most FAs in wheat are bound to indigestible polysaccharides, restricting its release. Recent *in vivo* findings, however, indicate that the fermentation of arabinoxylans by gut microbiota, which releases ferulic acid and butyric acid, has a positive impact on host health and metabolism [42,103]. Therefore, a knowledge gap remains regarding the interaction of polyphenols and AX in foods developed from different raw materials, such as BSG.

## 4. Health Benefits of Ferulic Acid, p-Coumaric Acid, and Arabinoxylans

The main bioactive compounds in brewer’s bagasse are ferulic acid, p-coumaric acid, and arabinoxylans. Ferulic acid has antioxidant, neuroprotective, and antidiabetic properties, similar to p-Coumaric acid, and exhibits antioxidant, anti-inflammatory, and hepatoprotective effects. In addition, arabinoxylans, a polysaccharide, act as soluble dietary fibers with prebiotic properties, promoting intestinal health and potentially mitigating chronic non-communicable diseases (Table 2).

### 4.1. Ferulic Acid

Ferulic acid (4-hydroxy-3-methoxycinnamic acid, FA) is a phenolic compound found in a wide variety of cereals and vegetables, such as broccoli, carrots, rice bran, corn, and barley [122,123,124,125,126,127]. The biosynthesis of FA in plants is a result of the shikimate pathway, which utilizes L-phenylalanine and L-tyrosine as key components. The conversion of these amino acids into cinnamic and p-coumaric acid is facilitated by enzymes such as phenylalanine ammonia lyase and tyrosine ammonia lyase. Further reactions, including hydroxylation and methylation, lead to the formation of FA [128].

The purified FA has been linked to several health benefits, and research has suggested that it possesses antioxidant activity, inhibits the neurotoxic aggregation of amyloid Aβ peptide (related to Alzheimer’s disease, and has antidiabetic properties [129,130,131,132]. Additionally, FA is a popular precursor substrate for vanillin production through microbial conversion by bacterial species and some fungi [133] and is used as a cosmetic ingredient to provide anti-aging, anti-wrinkle, anti-pigmentation, anti-inflammatory, and antimicrobial effects [130]. Regarding their high antioxidant capacity, it is mediated by different forms of actions, being a direct scavenger of free radical molecules or activating the expression of genes that code for antioxidant enzymes such as superoxide dismutase (SOD), glutathione s transferase (GST) and catalase (CAT) through the signaling pathway of Kelch-like ECH-associated protein-1 and nuclear factor E2-related factor-2 (Keap1-Nrf2) [134,135,136].

The impact of the treatment with FA in cellular cultures, model animals, and clinical trials has been tested in various research. In the first place, cellular models have shown the potential for treatment with FA improving lipid metabolism. In 3T3-L1 cellular lines, 10 µM FA downregulates peroxisome proliferator-activated receptor ϒ (PPAR-ϒ), and fatty acid synthase (FAS) expression, consistently with the induction of the lipolytic process [137]. In hepatocyte carcinoma HepG2 culture cells, treatments with 20 µM FA decrease reactive oxygen species (ROS) production and malondialdehyde (MDA), and increase the glutathione (GSH) and SOD enzyme levels in cells incubated with hydroxyquinoline, reducing the iron-induced HepG2 cell damage [106].

Ferulic acid (FA) has demonstrated significant protective effects in various animal models, particularly in mitigating oxidative stress and liver damage. For instance, a concentration of 75 µM FA can prevent the damage induced by ROS in a model of anoxia/reoxygenation and subsequently apoptosis in rat renal tubular epithelial cells [138]. Methyl ferulic acid is capable of increasing the gene expression of antioxidant enzymes such as SOD and GST, protecting the liver from the alcohol damage, through the activation of NOX4-ROS-MAPK, AMPK/ACC and PI3K/AKT signaling pathways, in C57BL/6 mice, a mouse model with a notably high alcohol preference [110]. Another study in rats indicated that FA mitigated oxidative stress via stimulating Nrf2/HO-1 signaling and inflammation, through the reduction in serum tumoral nuclear factor alpha (TNF-α) and interleukin (IL-1β), as well as hepatic (nuclear factor kappa B) NF-κB p65 [105]. The administration of FA restored free radical scavenging ability, reducing lipid hydroperoxide, protein carbonyl, and lipid peroxidation levels after 15 and 30 days of therapy in a rat model with liver injury induced by cadmium [139]. Indeed, there are several liver injury models in which FA administration decreases the histological damage findings and normalize the transaminase levels. In this regard, it is well-known that tetrachloromethane induces hepatotoxicity characterized by the leakage of liver enzymes such as aspartate aminotransferase (AST) and alanine aminotransferase (ALT) into the bloodstream. Treatment with methyl ferulic acid has a protective role in the liver in a dose-dependent manner, increasing the antioxidant enzyme activities, reducing MDA levels, improving histological alterations, and decreasing serum ALT and AST activities [107]. Administering 0.25 μmol/kg of liposomes encapsulating ferulic acid reduces AST and ALT by 35% and 30%, respectively [86]. Methotrexate is a folic acid antagonist with hepatotoxic effects [107]; however, treatment with FA (50–100 mg/Kg) decreases transaminases serum levels and increases SOD and glutathione peroxidase (GPx) activity [108]. Nevertheless, the most frequent stimulus for hepatotoxicity is the metabolic dysfunction associated with liver disease (MASLD), a metabolic condition in which insulin resistance and liver steatosis are present [140]. Supplementation with 100 mg/kg/day of FA significantly reduces liver size and hepatic steatosis, and improves insulin sensitivity and glucose tolerance in high-fat diet-fed mice [111]. Another study in mice shows even lower effective doses; 25 and 50 mg/kg could reverse cholesterol and triglycerides and hepatic content, reducing gene expression of lipogenic genes [141]. Moreover, the combination of exercise protocol and FA administration decreases ROS and MDA, as well as hepatic triglyceride (TG) concentration, compared to the effect of exercise alone [141].

**Figure 1:**
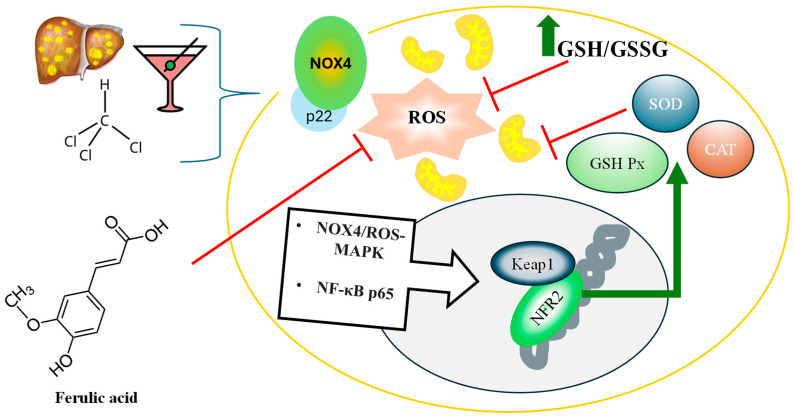
Mechanisms of ferulic acid mitigate oxidative stress during liver injury. Red lines indicate inhibition of oxidative stress. Green arrows indicate upregulation of gene expression and activity of antioxidant enzymes. The figure is the author’s creation. The overall layout and composition were designed and assembled using Microsoft PowerPoint (version 2512 from Microsoft 365). Chemical structure: [142].

Cellular cultures, model animals, and clinical trials indicate that FA demonstrates a wide range of health benefits, including antioxidant, neuroprotective, antidiabetic, and anti-inflammatory properties. Overall, FA holds promise as a therapeutic agent for managing oxidative stress-related conditions, liver diseases, and metabolic dysfunctions, warranting further investigation in clinical trials to fully elucidate its benefits and therapeutic potential. Ferulic acid (FA) has been reported to mitigate the generation of reactive oxygen species (ROS) produced by the NOX4 enzyme complex and dysfunctional mitochondria [134]. Injury-induced activation of pathways such as MAPK and NF-κB can lead to oxidative stress [105]. FA enhances the cellular antioxidant response by increasing the ratio of reduced glutathione (GSH) to oxidized glutathione (GSSG). The Nrf2-Keap1 pathway plays a crucial role in upregulating the gene expression program for antioxidant enzymes, including superoxide dismutase (SOD), catalase (CAT), and glutathione peroxidase (GSH-Px). These enzymes are instrumental in neutralizing ROS-associated liver damage caused by steatosis or drug exposure [105,136] (Figure 1).

### 4.2. p-Coumaric Acid (p-CA)

4-Hydroxycinnamic acid, or p-coumaric acid (p-CA), is a phenolic compound found in fruits and vegetables [143,144,145,146], and mushrooms [147]. This polyphenolic compound has been detected in extracts from brewery spent grains [31]. It is biosynthesized from tyrosine and phenylalanine through the shikimate metabolic pathway [148]. Studies show that p-CA has antioxidant, anti-inflammatory, anticancer [114], neuroprotective properties [149,150], among others.

p-CA exhibits significant protective effects against various forms of oxidative stress and metabolic dysfunction, as demonstrated in both *in vitro* and *in vivo* studies. In cell lines, there is robust evidence regarding its antioxidant capacity, for example, in HepG2 cells, KBrO3-induced oxidative stress, lipid peroxidation, and DNA damage were attenuated by p-coumaric acid, while Ras/Raf/MEK signaling and apoptosis were suppressed [113]. Hepatoprotective effects were also observed in treated p-CA (10–20 µM), reducing lipid accumulation, and enhancing lipolysis and β-oxidation in HepG2 cells. Additionally, cyclooxygenase 2 (COX-2) gene expression and Prostaglandin E2 (PGE2) accumulation were inhibited, suggesting a protective mechanism against palmitate-induced hepatocyte injury [112]. Metabolic improvement is also demonstrated in other cell lines. In L6 skeletal muscle cells, p-CA activated AMPK in a dose-dependent manner, promoting fatty acid β-oxidation and glucose uptake while reducing triglyceride accumulation. These effects were counteracted by AMPK inhibition, suggesting a role in metabolic regulation [151]. These results on liver and muscle cells suggest that coumaric acid may influence the metabolic response of different tissues.

In animal models, p-CA enhances the expression of key antioxidant enzymes and factors, providing hepatoprotective effects against chemically induced liver damage, such as that caused by cisplatin, carbon tetrachloride, bile duct ligation, and fipronil exposure. p-CA protects against cisplatin-induced hepatotoxicity in Wistar rats [152]. p-CA reduced liver necrosis and cholestasis markers in male Wistar rats subjected to carbon tetrachloride intoxication and bile duct ligation [117]. The hepatoprotective effects were confirmed through biochemical and histological analyses. Additionally, p-CA mitigated fipronil-induced liver damage by reducing oxidative stress, inflammation, and structural liver alterations [114]. In C57BL/6J mice on a high-fat diet, p-CA decreased adiposity, non-alcoholic fatty liver disease (NAFLD), and metabolic dysfunctions by lowering white adipose tissue weight, adipocyte size, and plasma leptin levels, while enhancing fatty acid oxidation and fecal lipid excretion. Administration of 100 mg/Kg p-CA improved lipid metabolism in high-fat diet (HFD)-induced mice. Its effects were associated with increased Nrf2-related antioxidant enzyme expression and reduced lipid peroxidation, suggesting potential therapeutic benefits for hyperlipidemia [153]. In line with obesity-related metabolic dysfunction, p-CA acid reduced adiposity, hepatic lipid accumulation, and inflammation in HFD-fed mice without affecting food intake or body weight. These effects were linked to decreased lipogenesis, increased fatty acid oxidation, and enhanced fecal lipid excretion. Additionally, insulin resistance and inflammatory markers were significantly lowered [115]. p-CA mitigated liver damage induced by dust and ischemia–reperfusion in rats by reducing oxidative stress, inflammation, and autophagy [116].

Another hepatoprotective effect has been described, exhibiting strong antioxidant and anti-inflammatory properties against LPS/D-GalN-induced liver injury in rats [154]. Liver function markers, lipid profile, and oxidative stress parameters were improved and TNF-α, IL-6, and IL-1β levels were reduced. The antioxidative role of p-CA was also described in rat kidney, where administration of p-CA for 7 days reverted the kidney damage gentamicin-associated MDA levels and inflammation [155].

More evidence related to beneficial metabolic effects of p-CA has been described. In the HFD-fed model supplemented with 120 mg/kg of p-CA, it has been observed that an increase in the secretion of peptide-like glucagon-1 (GLP-1), as well as a decrease in intrahepatic lipids through the actions of nucleophosmin 1 (NPM1), glucose-regulating protein 78 (GRP78), and calcium, diminish the effect of metabolic dysfunction-associated steatotic liver disease (MASLD) [156]. p-coumaric acid (p-CA) has been studied for its ability to reduce lipid accumulation *in vivo* and *in vitro*. It was observed that the expression of several lipases and genes involved in fatty acid oxidation increased, promoting the AMP-activated protein kinase phosphorylation. These effects, mediated by peroxisome proliferator-activated receptor (PPAR) activation, suggest that p-CA is a lipid metabolism regulator with therapeutic potential [157]. In the same line, in rats with chronic kidney disease (CKD), p-CA was administered, resulting in increased muscle weight and area. Levels of pro-inflammatory cytokines were reduced, and antioxidant enzyme activity such as SOD, CAT and glutathione peroxidase (GSH-Px) was enhanced. This effect was mediated by the inhibition of the Toll-like receptor 4/myeloid differentiation protein 88/nuclear factor kappa B (TLR4/MyD88/NF-κB) pathway, which led to the attenuation of muscle atrophy [158]. These studies suggest that the metabolic effect of p-CA is associated with action in the liver and other tissues.

**Figure 2:**
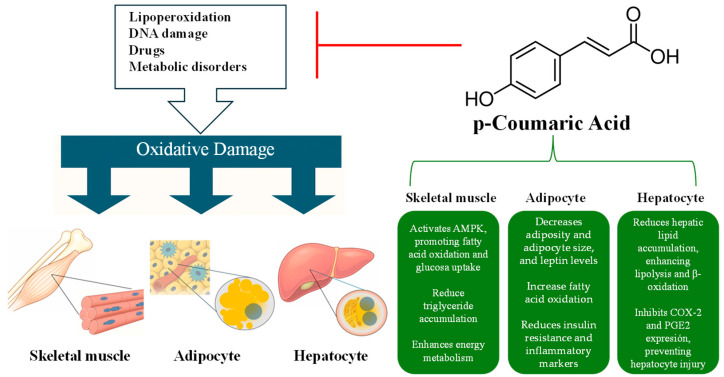
Protection against oxidative damage in metabolic tissues by p-coumaric acid. Red line indicates inhibition of oxidative stress. Blue arrows indicate oxidative damage on different tissues. Green blocks indicate antioxidant effects of p-CA. The figure is the author’s creation. The cell and tissue vector graphics were generated using an AI language model (ChatGPT version 5.2). The overall layout and composition were designed and assembled using Microsoft PowerPoint (version 2512 from Microsoft 365). Chemical structure [159].

These findings collectively suggest that p-CA has a broad spectrum of beneficial effects, potentially making it a valuable therapeutic agent for addressing oxidative stress, liver injuries, and metabolic disorders through its antioxidant and anti-inflammatory properties. p-Coumaric acid has been identified as a protective agent against oxidative damage induced by lipoperoxidation, DNA damage, medications, and metabolic disorders [114]. Oxidative stress, which adversely affects skeletal muscle, adipose tissue, and the liver, is a key contributor to metabolic dysfunction [115,117]. p-CA mitigates this damage by enhancing fatty acid oxidation, reducing lipid accumulation, and modulating inflammation across various tissues. The molecular structure of p-CA is linked to its tissue-specific effects, highlighting its potential therapeutic value in managing metabolic disorders [117]. Collectively, these findings underscore the promise of p-CA as a treatment option for metabolic dysfunctions (Figure 2).

### 4.3. Arabinoxylans (AX)

The AX are polysaccharides found in the hemicellulose component of plant cell walls [71]. They are present in high amounts in cereal grains such as wheat, barley, oats, corn, and rice, making up 67–71% of the walls of the aleurone layer in barley and 20% of cell walls [71]. The arabinoxylans are side chain-branched heteroglycans composed of pentose sugar, especially xylose and arabinose, with some arabinose side chains carrying esterified ferulic acid residues; therefore, for a different cereal, such as wheat and barley, arabinose can be further cross-linked to ferulic acid at the C5 position via an ester linkage [71,100,160]. As defined by the Codex Alimentarius Commission (CAC, 2009) [161], they are considered as dietary fiber due to their carbohydrate polymer composition of ten or more monomeric units, which cannot be hydrolyzed by the endogenous enzymes of the human small intestine. Arabinoxylans have been shown to have various physiological benefits, including an effect on immune response, anti-tumor activity, increased fecal volume, benefits in patients with glucose intolerance, and prebiotic properties [90,91], making them a product of interest for the formulation of functional foods. Arabinoxylans could be considered prebiotic substances, as their carbohydrate structures are fermented by beneficial bacteria in the gut, such as bifidobacteria and lactobacilli species [93,162].

The primary nutritional benefit of AX is their function as soluble dietary fibers, which can enhance gut microbiota health. When hydrolyzed, AX produce prebiotics such as arabinoxylan–oligosaccharides (AXOS) and xylooligosaccharides (XOS). Due to their complex molecular structure, they can reach the colon largely intact. In the colon, *Bifidobacterium* species ferment these fibers, producing short-chain fatty acids (SCFAs) like acetate, propionate, and butyrate. These SCFAs are fermentation products that contribute to gut homeostasis [88] and offer additional health benefits, such as reducing inflammation and enhancing the integrity of the intestinal epithelial barrier [89,90,91]. Although the symbiotic effect of AX and *Lactobacillus fermentum* HFY06 has been related to the potential to prevent and treat colitis in mice, increasing the body’s antioxidant capacity and reducing inflammation [163], the association of arabinoxylans with Lactobacillus is less well-known.

AXOS derived from wheat bran have been shown to mitigate metabolic issues induced by a Western diet in mice, improving conditions like liver steatosis, high cholesterol, and glycemia [92]. These beneficial effects are mainly due to their ability to stimulate the growth and activity of Bifidobacteria in the gut [88]. Therefore, the AXs promote digestive benefits by improving intestinal regularity through growing bacteria in the intestines [164,165]. Various mechanisms of action for arabinoxylans have been identified, including the enhancement of the intestinal epithelial barrier in overweight and obese individuals [93]. Supplementation has been associated with improvements in gut permeability and the expression of tight junction proteins [94].

On the other hand, some evidence shows that there are many beneficial effects on chronic non-communicable diseases. In the first place, animal studies are developed using different sources of AX; arabinoxylan from *Plantago asiatica* L. reduces metabolomics urine markers in diabetic rats [166]. In addition, treatment with 6% of AX reduces triglycerides and lipoperoxidation markers, and also increases both SOD and GSH-PX activity in HFD-fed rats [118]. Arabinoxylan supplementation in diabetic rats increased fiber-degrading bacteria, leading to higher short-chain fatty acids and reduced levels of opportunistic pathogens and 12α-hydroxylated bile acids, while increasing indolepropionate and eicosadienoic acid [166]. Rice bran AX, a by-product of rice milling, significantly reduced high-fat diet-induced obesity by decreasing lipid accumulation and regulating hepatic fatty acid metabolism genes, normalizing gut microbiota composition, increasing beneficial bacteria, and decreasing inflammatory biomarkers such as TNF-α and IL-6 [111]. In the same line, the anti-inflammatory effect has been documented in a hepatitis mouse model, where a downregulation of interleukin 18 and a reduction in NF-κB and JNK phosphorylation were observed following supplementation with a modified arabinoxylan derived from rice bran [167]. The immunomodulatory effect of AX has been described, but the exact mechanism involved is not clear yet. It has been proposed that the structural similarities between AX and lipopolysaccharides (LPS) could stimulate an immunological response by activating receptor TLR4 [168]. Therefore, AX not only supports intestinal health, promoting beneficial gut bacteria, but also potentially helps mitigate chronic non-communicable diseases.

**Figure 3:**
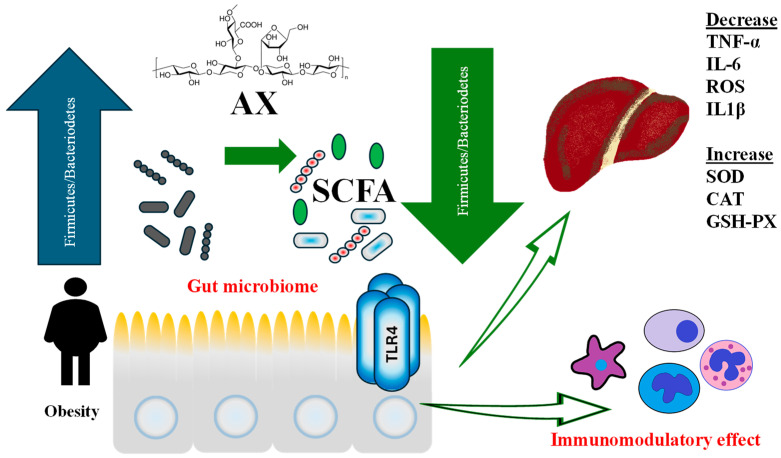
Mechanisms of arabinoxylans in restoring gut health and reducing inflammation in individuals with obesity and metabolic disorders. Blue arrow indicates negative effect on gut microbiome. Green arrows indicate positive effect on gut microbiome, immune response and liver. The figure is the author’s creation. The cell and tissue vector graphics were generated using an AI language model (ChatGPT 5.2). The overall layout and composition were designed and assembled using Microsoft PowerPoint (version 2512 from Microsoft 365).

Regarding liver health, finger millet arabinoxylan supplementation prevents weight gain, improves glucose tolerance and lipid profile, and increases the gene expression of genes such as Fatty Acid Synthase (*FASN*), Acetyl-CoA Carboxylase (*ACC*), Sterol Regulatory Element-Binding Protein 1c (*SREBP-1c*), and 3-Hydroxy-3-Methylglutaryl-Coenzyme A Reductase (*HMGCOA-R*) in mice with high-fat diet (HFD)-induced obesity [169]. A few reports exist about the effectiveness of AX on human health; one of these demonstrated that intake of rice bran AX compound improves the metabolic and oxidative biomarkers associated with adults with non-alcoholic fatty liver disease (NAFLD), such as GGT and 4-hydroxynonenal, both of which decrease in these patients after a 90-day supplementation (1 g/day total) [119]. Its effectiveness on metabolic diseases such as diabetes has also been studied. Treatment of diabetic mice during 4 weeks with AX waste derived from wheat gluten protein production decreased the fat infiltration of the liver and improved metabolomics markers related to liver lipid accumulation in type 2 diabetics [111]. Therefore, supplementation of AX from sources like finger millet and rice bran has shown potential in improving liver health and metabolic profiles, including after HFD. Obesity is associated with gut dysbiosis, a condition that can potentially be ameliorated through the consumption of AX. Arabinoxylans have been shown to increase the Firmicutes/Bacteroidetes ratio and promote the synthesis of short-chain fatty acids (SCFAs) [111]. These SCFAs interact with the TLR4 receptor in the gastrointestinal tract, leading to immunomodulatory effects that indirectly reduce pro-inflammatory markers such as TNF-α, IL-6, ROS, and IL-1β in the liver. Additionally, this interaction enhances antioxidant defenses, including superoxide dismutase (SOD), catalase (CAT), and glutathione peroxidase (GSH-PX) [167,168] (Figure 3).

Although most animal studies have not demonstrated a specific molecular mechanism of action, AX are probably performing these effects through modification of the gut microbiota environment. Indeed, administration combined with β-glucan and xyloglucan improves lipid metabolism by regulating bile acid metabolism. In addition, AX treatment decreases the Firmicutes/Bacteroidetes ratio and Proteobacteria, llobaculum, Bifidobacterium, and Faecalitalea presence, showing beneficial effects on the microbiota [120]. On the other hand, some approaches have been assessed in clinical trials; diet supplementation with 1 g per day of polysaccharide from rice bran arabinoxylan can decrease several biomarkers associated with non-alcoholic fatty liver disease in a 90-day randomized, double-blind, placebo-controlled trial [119]. Diet supplementation with bread and muffins with 14% AX-rich fiber significantly reduced fasting blood glucose and fructosamine concentrations in a sample of 15 diabetic persons, respecting the control subjects [121].

The food industry has the potential to develop bioactive structures from AX. For instance, Biobran is a denatured hemicellulose, primarily composed of AX, derived from rice bran through the enzymatic action of shiitake mushrooms. Administering Biobran to a mouse model of Alzheimer’s disease resulted in a significant increase in glutathione (GSH) levels and a reduction in both malondialdehyde (MDA) levels and the number of amyloid plaques in the brain [170]. However, more background information needs to be gathered to understand the role of arabinoxylan supplementation to aid the restoration of cognitive function.

Interestingly, it has been reported that AX undergoes fermentation by gut microbiota, resulting in the production of ferulic acid and butyric acid, which enhance host immunity [42]. Ferulic acid and SCFAs, produced from the microbial fermentation of cereal-derived arabinoxylans, interact to influence host health and metabolism [103]. Although both ferulic acid and SCFAs are recognized as having beneficial effects, the integrated molecular mechanisms explaining how these compounds act together to regulate intestinal health and host metabolism are not entirely clear. On the other hand, the different processes to produce an ingredient from BSG can affect the release of ferulic acid produced by the gut microbiota.

## 5. Challenges in Food Ingredient Development from BSG

The processes employed to prepare fruits and vegetables—such as cooking, freezing, and various biotechnological treatments—can influence both the quantity and quality of bioactive molecules as well as their availability [171]. Stabilizing raw materials is essential because many agro-industrial residues exhibit very high humidity levels (>75%) and water activities above 0.6, conditions under which available carbohydrates can foster microbial growth. For instance, in beer bagasse, microorganisms such as yeasts, fungi, lactic acid bacteria, aerobic bacteria, and even clostridia have been detected [44]. By reducing humidity and water activity through processing, it is possible to stabilize the residue while still preserving its functional properties. However, it is important to note that stabilization—particularly through dehydration processes—often requires high temperatures, which may compromise the bioactivity of sensitive compounds like antioxidants due to their inherent lability [172,173].

In recent decades, research has increasingly focused on the health benefits of various foods, emphasizing the presence and pure-state bioactivity of bioactive compounds. Yet, the intrinsic bioactivity of these molecules does not always translate into measurable benefits when they are incorporated into a food matrix. Therefore, understanding their bioavailability is crucial for developing strategies that improve consumer well-being through dietary interventions [174,175]. Factors such as low solubility, instability in gastric juices, and challenges with intestinal absorption hinder the effectiveness of many bioactive compounds [176]. Additionally, the digestive process can further modify the availability of these molecules [177,178,179].

Bioavailability refers to the proportion of a compound that is absorbed and reaches target tissues, either intact or metabolized, after ingestion. It is determined by two interrelated stages: bioaccessibility (the fraction of a compound that is released during digestion) and bioactivity (the capacity of the absorbed compound to exert a biological effect). Although few studies have directly assessed the bioavailability of polyphenolic compounds in BGS, indirect evidence of their absorption has been observed via the detection of these compounds in blood plasma following the consumption of polyphenol-rich foods such as apple or black currant juice, tea, and red wine in rats and humans [180,181,182]. The following sections discuss the processing methods applied to brewer’s bagasse that can affect the content and composition of its bioactive compounds, as well as the challenges involved in preserving these compounds without compromising the valuable fiber content.

### 5.1. Stabilization of Beer Bagasse

Proper dehydration of bagasse is essential for storage, as it minimizes microbial growth and enables the production of safe ingredients from agro-industrial wastes. Notably, the dehydration method plays a critical role in determining the final composition; for example, freeze-drying results in significantly higher lipid and protein contents and lower carbohydrate levels compared to oven drying at 45 °C and 105 °C [4]. Moreover, vacuum microwave drying produces baked chips with moderate protein functionality (including water and oil retention capacities) and the highest overall acceptability when compared to chips made via freeze-drying or oven drying at temperatures up to 70 °C [183]. However, dehydration at high temperatures can adversely affect the stability of phenolic compounds. It has been demonstrated that the antioxidant capacity of eight phenolic acids—including ferulic acid—decreases linearly as temperatures increase from 90 °C to 150 °C. However, the rate of decline varies among the compounds [184].

Few studies have examined the influence of dehydration on both the concentration and quality of phenolic compounds with antioxidant activity in brewer’s spent grain (BSG). The comparation of intermittent infrared (IR) drying—performed at temperatures ranging from 350 °C to 380 °C over 41 min—with a forced convective oven process (hot air) at 85 °C for 2 h revealed significant differences in the final moisture content and protein levels, while ash and lipid contents remained similar across the methods [185]. Furthermore, the levels of phenolic compounds (746.57 ± 66.28 to 811.09 ± 81.52 mg GAE/kg dry base (d.b.)) and their antioxidant capacity, as determined by the DPPH method (1269.60 ± 28.11 to 1413.30 ± 174.68 mg Trolox/kg d.b.), did not differ significantly, suggesting that these bioactive components remain relatively stable despite variations in moisture and protein content. Regarding ferulic acid—the predominant phenolic compound in BSG—it has been reported that raising the processing temperature from −173 °C to 726 °C increases the molecule’s enthalpy, heat capacity, and entropy [186]. This enhanced vibrational activity results in the instability and degradation of ferulic acid. Therefore, choosing the appropriate dehydration method is essential to balance the preservation of bioactive compounds and the overall quality and functionality of the resulting agro-industrial ingredients.

### 5.2. Innovative Processing for the Full Use of BSG

Cereals have been reported to exhibit low bioavailability of ferulic acid (FA) because FA is strongly bound to the fiber fraction via cross-linking with arabinoxylans and lignins [122]. Consequently, the composition of the matrix severely limits FA bioavailability, highlighting the need to evaluate processing methods that maintain matrix stability without compromising the bioavailability of bioactive compounds. In this regard, it is necessary to consider that the digestion process strongly influences the bioaccessibility and bioavailability of the bioactive compounds of a plant matrix. For example, in the specific case of brewer’s bagasse, it has been observed that the *in vitro* digestion of breads made with brewer’s bagasse does not have a significant effect over oxidative stress and anti-inflammatory processes in Caco-2 cells [187], while the availability of minerals and B-group vitamins during simulated digestion has been investigated, noting variations based on the raw material [45]. Despite these advances, further research is required to determine how different processing methods affect the bioavailability of antioxidants from brewer’s bagasse.

While hydrolysis effectively extracts antioxidants, it does not allow the utilization of the residual fiber. In contrast, bioprocessing methods employing enzymes and microorganisms have been applied to enhance the recovery of antioxidant compounds from plant matrices such as seeds, wines, juices, and tea. These techniques could optimize the use of BSG by improving its digestibility while preserving both the antioxidants and the fiber. Enzymatic treatments, for instance, offer an eco-friendly and energy-efficient alternative to alkaline extraction because they can produce fractions with varied chemical, functional, and technological properties without damaging valuable molecules. Feruloyl esterase (FAE, EC 3.1.1.73) has been used to hydrolyze the ester bonds linking ferulic acid and cell wall polysaccharides in wheat bran [188]. Additionally, xylanases—mainly from glycoside hydrolase families 10 and 11—can break the β-(1,4) bonds between xylopyranoside residues, partially solubilizing water-insoluble AX and depolymerizing water-soluble AX [189,190]. These findings demonstrate that bioprocesses can release both ferulic acid and AX.

Commercial enzymes with proteases, cellulases, tannases, and hemicellulases activities from fungal sources (such as *Aspergillus* sp. and *Trichoderma* sp.) have been shown to increase the recovery of antioxidant compounds by up to 80% from different by-products such as grape pomace, chicory, fennel and duckweed, among others [191,192,193,194]. On the other hand, it is also possible to use commercial enzymes derived from bacteria, such as Alcalase protease (from *Bacillus licheniformis* bacteria), which allows for increased recovery of compounds with antioxidant activity from agro-industrial waste [195]. These enzymes can break down plant cell walls, releasing or producing bioactive compounds [196]. Moreover, bacterial fermentation, with *Lactobacillus strains*, has enhanced the recovery of enzymes such as feruloyl esterase, tannase, and phytase, which are involved in the hydrolysis and release of antioxidant molecules [197,198,199,200,201,202,203].

The recovery yields of bioactive compounds are influenced by multiple factors, regardless of whether enzymatic or fermentative processes are employed. In the first case, factors such as the type of catalyst and its origin, the processing time, the enzyme/substrate ratio, pH and process temperature, and the structure of the substrate or raw material will influence both the yield and the biological activities of the product; specifically, raw material structure affects enzymatic hydrolysis by determining enzyme–substrate binding accessibility through specific active sites for catalysis determined by the accessibility of the enzyme to its substrate [194,204,205].

In fermentation processes, several factors influence the production and release of bioactive compounds. Key parameters include the type of microorganism, process duration, and temperature, which are crucial for metabolic activity and enzyme production. Additionally, the characteristics of raw materials or substrates, medium pH (which regulates enzyme activity), and aeration conditions (determining whether fermentation is aerobic or anaerobic and affecting enzyme activity in the medium) significantly impact compound production. The fermentation system—whether submerged or solid-state, aerobic or anaerobic—along with the specific microorganism used, determines the type of enzymes produced: membrane-bound, intracellular, or extracellular. These enzymes are responsible for the hydrolysis and transformation of molecules, ultimately facilitating the release of bioactive compounds into the medium [204,205].

In BSG, several studies have reported increases in antioxidant, protein, and oligosaccharide compounds following enzyme treatment with pectinases, xylanases, glucuronidases, feruloyl esterases, among others [206,207,208], as well as the production of valuable products—including organic acids, amino acids, volatile fatty acids, enzymes, vitamins, and second-generation biofuels—through raw material fermentation [208,209]. During fermentation, microorganisms produce enzymes that hydrolyze macromolecules, thus releasing various compounds. Likewise, this process enables the transformation of molecules to produce different bioactive products, thereby increasing the final product’s nutritional value and bioactivity [205]. For example, the solid substrate fermentation (SSF) of BSG with microorganisms such as *Rhizopus oligosporus* has improved the digestibility of the raw material, reduced the presence of antinutritional compounds, and facilitated the degradation of complex carbohydrates, increasing the bioavailability of various nutrients that have antioxidant, anti-inflammatory and prebiotic properties, among others [210]; whereas, in the case of using *Pleurotus ostreatus* (an edible mushroom), there is a significant reduction in the dry mass of the BSG, a 66% increase in protein content, and a decrease in lipids and carbohydrates, as well as phytic acid (antinutrient), but an increase in the presence of phenolic compounds [211].

On the other hand, processes developed with different fungal strains such as *Aspergillus* spp., *Bacillus* spp., and *Rhizopus* spp. have promoted greater recovery of proteins and amino acids from BSG, and an increase in the presence of phenolic compounds and their antioxidant activity [212]. Specifically, the use of fermentation processes with microorganisms such as *Escherichia coli*, *Aspergillus* sp., *Saccharomyces cerevisiae*, *Debaryomyces hansenii*, *Lactobacillus rhamnosus*, *Bacillus* sp., *Penicillium* sp., and *Trametes versicolor* on pretreated (or pre-hydrolyzed) BSG has allowed us to obtain products such as citric acid, lactic acid, or enzymes such as alpha amylase, cellulases, xylanases, laccase, among others, either through submerged or solid substrate fermentation [208,213], further highlighting the potential of fermentation processes for the valorization of spent grain through the recovery of different compounds.

Fermentation processes also allow the increase in nutritional and technological quality of BSG for use as a food ingredient [214,215]; evaluation of the effect of controlled fermentation of BSG with a patented process using two types of lactobacillus strains on bread and pasta production observed the obtention of a BSG with lower fat content but higher dietary fiber content, which consequently generates for bread an increase in specific volume, a reduction in crumb hardness, less microbial growth over time, and a lower rate of sugar release during *in vitro* digestion tests of the product; whereas, in the case of pasta, products with a higher content of dietary fiber are obtained, and a theoretical (predicted) glycemic index lower than that of the control pasta, especially when the fermented brewer’s bagasse was used. Other authors report that fermentation processes can affect the antioxidant activity of plant-based foods by releasing and producing bioactive compounds [196].

On the other hand, the effect of fermentation time with baker’s yeast (*Saccharomyces cerevisiae*) has been studied to produce bread with brewer’s spent grain [216], observing a significant effect on variables such as moisture, protein level (increasing), caloric value, and mineral content (increasing levels of calcium, magnesium, and phosphorus), without affecting the sensory evaluation of the product, especially when the level of wheat flour replacement with BSG flour is 20%, which implies that a fermentation process allows for an improvement in its nutritional and sensory quality.

In the context of valorizing brewer’s bagasse as a functional food ingredient, emerging technologies such as ohmic heating and microwave processing offer significant advantages. These methods can induce structural changes in the fiber that promote the release and assimilation of antioxidant compounds. For instance, ohmic heating has been shown to facilitate the efficient release of intracellular compounds and enhance extraction yields [217]. Studies with olive leaves [218] and grape pomace [219] have demonstrated that these techniques not only increase the extraction yield of bioactive compounds but also result in higher total phenol content and antioxidant activity compared to conventional heating. Additionally, it has been demonstrated that ohmic heating improved the bioaccessibility of phenolic compounds from tomato flours during *in vitro* gastrointestinal digestion, suggesting that this technique optimizes phenolic extraction and facilitates their subsequent assimilation [220].

In BSG, it has been described that hydro-ethanolic extracts obtained with 60% ethanol and ohmic heating exhibited the highest antioxidant activity—with 4-hydroxybenzoic acid being the predominant compound—and inhibited the growth and biofilm formation of pathogens such as *Listeria monocytogenes* [221]. Similarly, microwave pretreatment (MW-BSG) significantly increased levels of soluble dietary fiber and water-soluble proteins under specific conditions (540 W, 3 min, 1:5 material–liquid ratio), thereby enhancing the nutritional functionality and aroma of the product without compromising its overall quality [222]. Notably, one ton of prehydrolysate BGS with alkaline and microwave pretreatment has been shown to yield 133 kg of arabinoxylans for obtaining butanol [223]. Therefore, the mixtures of enzymatic treatment and microwaves can be combined for the optimization of AX production which is used as a food ingredient.

Given that the molecular structure of fiber influences its physiological effects—such as the release and intestinal absorption of antioxidants, as well as the selection of microbial species that utilize it as a growth substrate [224,225]—it is essential to understand fiber structure when developing strategies to develop ingredients higher in fiber with enhanced antioxidant bioavailability, but with organoleptic acceptance as a food ingredient.

**Figure 4:**
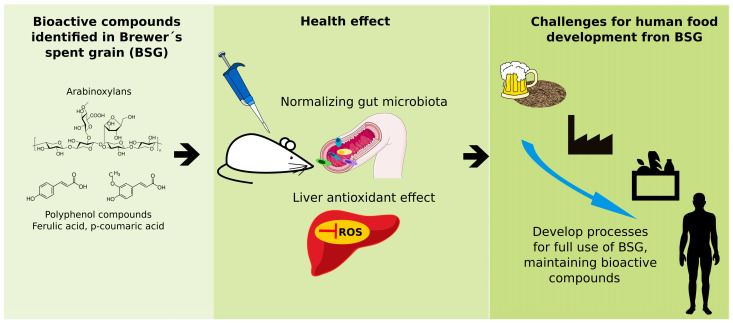
Potential of beer bagasse as a food ingredient. Different color blocks indicate stages necessary for food ingredients development. Red line indicates inhibition of oxidative stress. Blue arrow indicates necessity of process improvements. The figure is the author’s creation using Inkscape software version 1.1 [226]. Icons and chemical structures [227].

Future challenges include generating knowledge for waste processing to obtain ingredients that do not affect the organoleptic acceptance of the generated products and improving the assimilation of bioactive compounds at the intestinal level and their relationship with the intestinal microbiota, which is crucial for designing functional foods from by-products like brewer’s bagasse (Figure 4). Additionally, all processes—both traditional and emerging—must be evaluated for scalability and logistical feasibility to ensure industrial sector viability.

### 5.3. Sustainability and Circular Economy Benefits of BSG Valorization

The use of BSG as a functional food ingredient, along with other strategies (such as the production of animal feed and plastics), offers an opportunity to reduce waste. The brewing industry generates significant quantities of by-products, of which BSG constitutes approximately 85% [3]. According to data provided by the industry, a craft brewery generates approximately 2 tons of spent grain per month; 100 microbreweries amount to around 200 tons of this agro-industrial waste per month, and it is estimated that at least 50% of this spent grain is discarded.

Our studies in BSG suggest it has a high-value composition, with polyphenol contents close to 1000 mg/100 gDW, of which approximately 200 mg/100 g of DW correspond to ferulic acid, along with a high dietary fiber content (40–64%), including between 3 and 14% arabinoxylans (unpublished data). Through enzymatic extraction strategies, it is possible to recover approximately 50% of the soluble solids, yielding roughly 500 g of the extract with higher ferulic acid and arabinoxylan content per kilogram of spent grain (unpublished data). This opens the possibility of fully valorizing this residue as a functional ingredient. Considering that commercial high-fiber bread (approximately 10% fiber content) retails for around USD 4/kg in Chile (as example) as of February 2026, the complete valorization of brewer’s spent grain transforms a residue of low or no economic value into a raw material with commercial potential, simultaneously contributing to waste reduction and the development of circular economy models.

Converting BSG into functional food ingredients simultaneously tackles waste management challenges, enhances nutrition, stimulates economic growth, and advances sustainable practices, demonstrating its significant potential for creating a more sustainable and equitable food system. Environmentally, BSG reuse significantly would reduce landfill waste while promoting circular economy principles by converting brewing by-products into valuable food ingredients. Nutritionally, BSG would provide abundant dietary fiber, protein, and bioactive compounds such as polyphenols, which improve food nutritional quality and support public health by preventing metabolic syndrome and cardiovascular disease. This would enhance food security through alternative nutrient sources and expanded access to nutritious food options.

Economically, BSG valorization would generate new market opportunities within the food and beverage sector, particularly benefiting craft breweries and SMEs through innovation, job creation, and economic diversification. This strategy would optimize barley’s nutritional potential while creating additional revenue streams. Finally, in the social sphere, the adoption of BSG as an ingredient would strengthen community participation and environmental awareness through educational programs and collaborations between the industry, academia, and the community. These collaborations would facilitate knowledge sharing and streamline the logistics for BSG valorization, while coordinating efforts toward achieving the Sustainable Development Goals.

## 6. Conclusions

This review highlights the importance of adopting a multidisciplinary approach to optimize the use of brewer’s spent grain (BSG) as a functional food ingredient. This by-product is rich in polyphenols, antioxidants, dietary fiber, and protein, making it a promising candidate for preventing metabolic syndrome and cardiovascular disease. Bioactive compounds, such as ferulic and p-coumaric acids, offer significant health benefits, including antioxidant, anti-inflammatory, and prebiotic effects. The studies of each molecule indicate that these compounds protect the liver by increasing the expression of antioxidant enzymes and reducing inflammation and oxidative stress, while compounds such as AX improve bowel regularity by promoting the growth of gut microbiota. However, knowledge of the effect of bagasse as a whole food is still limited.

The recovery of antioxidants and arabinoxylans depends on stabilization and processing that modify the raw material, with enzymatic processes standing out, as they can recover up to 33% of soluble arabinoxylans. Therefore, a key challenge is improving the bioavailability and assimilation of these antioxidants and fibers, which are often limited by the complex food matrix. Innovative processing technologies are needed to enhance the release and absorption of these bioactive compounds, thereby enabling the production of foods.

Although the low bioavailability of ferulic acid is primarily due to its binding to arabinoxylan structures, recent studies highlight the beneficial role of the gut microbiota in fermenting these compounds. This intestinal fermentation process releases ferulic acid and produces butyric acid, both of which have positive effects on host health and metabolism. Despite these advances, further research is needed to understand the interactions between polyphenols and arabinoxylans in BSG in the gut, the effects of BSG as a food on the liver, and the role of BSG in metabolic syndrome.

Furthermore, additional research is required to understand the health benefits of BSG consumption fully and to optimize processing methods that preserve its nutritional and functional properties. Addressing these challenges will be crucial for effectively incorporating BSG into the human diet as a sustainable, health-promoting ingredient, thereby contributing to waste valorization and sustainable food innovation. The study of the manufacturing scale-up and their logistical feasibility, combined with research into their biological effects on highly prevalent diseases, offers a broad field of study and the opportunity to evaluate the effects of BSG and other by-products in the population.

## Figures and Tables

**Table 1:** Nutritional composition and energy value of dried malt bagasse bran.

Nutritional Composition	Content Range (%)
Energy (Kcal)	248 [32]
Moisture	4.4–5.6 [32]
Minerals	2.7–4.6 [32]
Proteins	12.5–18 [32,35,36,38]
Fats	5.9–7.69 [32,35,36,38,43]
Carbohydrate	10–46.9 [32,35,38]
Fiber	40–48 [32,39,43]

This table presents a comprehensive overview of the nutritional composition and energy value of dried malt bagasse bran, as determined by various studies [32,35,36,38,43].

**Table 2:** Functional properties of ferulic acid, p-Coumaric acid, and arabinoxylans.

Compounds Name	Molecular Structure	Functional Properties
Ferulic acid, (2*E*)-3-(4-hydroxy-3-methoxyphenyl)prop-2-enoic acid	(CH_3_O)HOC_6_H_3_CH=CHCO_2_H 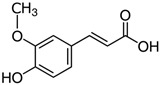	Decrease liver fibrosis [104]
		Decreases ROS production and hepatotoxicity drug-induced [105,106,107,108,109]
		Prevents liver steatosis and metabolic dysfunction-associated liver disease [110,111]
p-Coumaric acid (p-CA) or 4-hydroxycinnamic acid	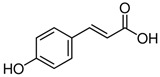	Hepatoprotective effects [112]
		Antioxidant and anti-inflammatory effect [113,114,115,116]
		Reduced liver necrosis and cholestasis [113,114,117]
Arabinoxylans, β-(1 → 4)-linked xylose units	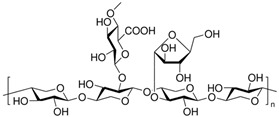	Increase lipolytic gene expression program [111,118]
		Decreases ROS production and increases antioxidant mechanism in liver [119]
		Improves intestinal microbiota and reduces glycemia and lipid profile [120,121]

## Data Availability

No new data were created or analyzed in this study.
